# Open source data reveals connection between online and on-street protest activity

**DOI:** 10.1140/epjds/s13688-016-0081-5

**Published:** 2016-05-06

**Authors:** Hong Qi, Pedro Manrique, Daniela Johnson, Elvira Restrepo, Neil F Johnson

**Affiliations:** Department of Physics, University of Miami, Coral Gables, FL 33126 USA; Department of Government, Harvard University, Cambridge, MA 02138 USA; Department of Geography, University of Miami, Coral Gables, FL 33124 USA

**Keywords:** open source data, online searches, civil unrest, social media

## Abstract

There is enormous interest in inferring features of human behavior in the real world from potential digital footprints created online - particularly at the collective level, where the sheer volume of online activity may indicate some changing mood within the population regarding a particular topic. Civil unrest is a prime example, involving the spontaneous appearance of large crowds of otherwise unrelated people on the street on a certain day. While indicators of brewing protests might be gleaned from individual online communications or account content (e.g. Twitter, Facebook) societal concerns regarding privacy can make such probing a politically delicate issue. Here we show that instead, a simple low-level indicator of civil unrest can be obtained from online data at the aggregate level through Google Trends or similar tools. Our study covers countries across Latin America during 2011-2014 in which diverse civil unrest events took place. In each case, we find that the combination of the volume and momentum of searches from Google Trends surrounding pairs of simple keywords, tailored for the specific cultural setting, provide good indicators of periods of civil unrest. This proof-of-concept study motivates the search for more geographically specific indicators based on geo-located searches at the urban level.

## Introduction

Improving understanding of the connection between online and real-world human behavior has important scientific and practical motivations [1–30]. The use of online media has gone from being a tool of communication [1, 2] to providing insights into a population’s collective thoughts and behavior [4–21]. In recent years, strong correlations have been found between variations in online activity and decision-making in complex systems such as financial market [7, 8], political protests [9–14], infection spreading [15, 16], and terrorist activity [17]. While advances in the understanding of the mechanisms and control of contagion processes in complex networks [3, 18–22] may describe driven collective action [23–30] (e.g. social unrest), the active monitoring of online activity provides real-time images of the current state of a system and potentially where it is heading. The likelihood that online activity may actually pre-empt on-street behavior is a tantalizing prospect [31]. However, even if online behavior merely reflects and hence reports on-street activity, this in itself can be very important in the absence of reliable media sources or in relatively remote locations. Among the human phenomena of interest, social instability is a particularly important one since it has long been a feature of state-society relations and also can lead to other problems such as widespread violence and insecurity [9, 29, 30]. Indeed socio-technical advances have created favorable conditions for forecasting certain types of mobilizations or protests while simultaneously generating large reservoirs of online data [31–33]. Such data offers a new avenue of research where information flows may provide fresh insight into human behavior. However while some information may in principle be obtainable from personal communications in the form of Tweets or the underlying Twitter accounts, or from inside informants, this level of specificity is not always readily available and can be politically dangerous because of concerns of snooping among the wider population.

Here we show that a simple and arguably less controversial examination of open source Google Trends searches can not only act to detect bursts of civil unrest, but may potentially pre-empt them. Such detection is useful in the absence of reliable media sources and avoids gathering any information about individuals, their Internet accounts, or their behaviors. The fact that online and on-street collective behaviors would be coupled in this way is consistent with the notion that civil unrest is a time-varying coordinated interaction between individuals, groups or populations with significant feedback between issues in the past and present. Our study considers protests across Latin America during 2011-2014 and grew out of a national project funded by IARPA (Intelligence Advanced Research Projects Activity) [31–33]. Specifically, we find that a combination of volume and momentum (i.e. rate-of-change) of particular keyword searches serves as a good detection signal, in contrast to just the search volume. Executing an exhaustive automated search over combinations of language and culture specific keywords, we find a preference based on performance and simplicity for pairs of keywords tailored to a specific country.

Having studied various options including Twitter and Flickr, we found Google Trends (GT) to be the most suitable data-source to use for reasons of reliability, documentation and geographic accessibility. Hence Google Trends forms the focus of this paper and is used to provide all the results presented. Since we are interested here in proof-of-concept, we also aggregate the data to the weekly scale - though this could again be generalized down to the daily scale using the same methodology as in this paper.

## Methods

In order to probe the potential relationship between online searches surrounding the topic of protests, and the actual occurrence of on-street protests, we proceeded using the following five step process. Our methodology for building the event dataset evolved from our participation in the 2011-2014 IARPA (Intelligence Advanced Research Projects Activity) national project on Open Source Indicators (OSI) [32, 33]. Latin America provides an ideal laboratory for this analysis since there is significant variation in search activity across countries, coupled with a substantial number of civil unrest events.

First, we obtained a list of protest events occurring in countries across Latin America starting in 2011, from the database that we helped build under the IARPA OSI project [31]. This database was compiled in real-time as protest events unfolded using reports from the top 100 newspapers in Latin America (see http://www.4imn.com/). Each reported event was scrutinized by an IARPA-funded team of academics from multiple institutions whose expertise included geopolitical issues and current affairs across Latin America, contentious politics, labor disputes, election campaigns, linguistics as well as native Spanish and Portuguese speakers with knowledge of idiosyncrasies in language use within each country. Several co-authors of the present paper were members of this academic team, as well as previous group members Nicolas Velasquez and Dr. Ana Morgenstern. The final decision to include events in the database was audited by an independent agency (MITRE Corporation) who themselves carried out independent checks. This dataset was officially termed the Gold Standard Record (GSR) by IARPA, in the expectation that it was as close to ground truth as possible given current technological capabilities, hence we continue to use the term GSR here. We have subsequently taken this GSR list and re-checked it in light of media and academic discussions that subsequently emerged concerning some of these events. We found no errors that change the results presented here.

Second, we investigated a variety of potential sources for extracting search volumes online for different keywords. We required a reliable yet popular source that would provide us with search volumes over time for specific words, and which was also from accounts operating in specific countries. These specifications reduced the potential sources to Twitter and Google Trends. We then carried out prediction tests as specified in this section using volume and momentum. Google Trends emerged as the superior choice, showing a 15% improvement in performance as compared to Twitter - hence we use Google Trends in all that follows in this paper.

Third, our team of native Spanish (and Portuguese) speakers assembled a dictionary of words associated with on-street protests in each of the different countries, around a variety of motives from employment through to education. These provide the list of keywords for each country, from which pairs of words are then taken to test the prediction method. One of the main challenges that we needed to overcome, was the variation in language - not just between Spanish and Portuguese, but also within the same language due to the subtle differences in terminology used across different media sources and countries. For example, the key word ‘paro’ in Spain is related to unemployment while in Latin America it relates more to a protest strike. Our overriding rule was to choose key words that are relevant to protests in the context of the different countries of Latin America. At the same time, there is of course no guarantee that simply by combining words that could be associated with a protest, the subsequent emergence of a positive signal in terms of volume and momentum is necessarily tied to a looming street protest.

Fourth, we explored exhaustively the combination of (1) keywords from searches in step 3, and (2) combinations of search volume and its derivatives, to see if we could maximize the association between online and on-street activity - in particular using the online activity as a predictor of the subsequent appearance of on-street protests. This was the most time consuming part of our study since (1) and (2) needed to be performed in tandem. The overall driving principle was to identify collections of keywords whose searches volumes and their derivatives gave the closest association with actual on-street protests. We did this in two complementary ways, both of which interestingly gave similar results in terms of performance. The performance measure is discussed later in this section. In the first, which we call here the ‘semi-automated’ method, we manually took pairs of words from the keyword catalog assembled by our team, and checked if their search volume at a given time-step gave a close association with on-street protests in the next time-step. Then we generalized this to include a combination of search volume at a given time-step, and the derivatives of search volume such as its rate-of-change over the past few time-steps. This revealed to us that the momentum (i.e. rate-of-change) was the most reliable derivative to use, since it was less noisy than higher derivatives. Since there is essentially never a time-step when there is zero search volume for any of the words, we introduced a threshold to help distinguish signal from noise. In particular, in order to make a prediction for an on-street protest event in the next time-step, we demanded that the volume of a particular search and rate-of-change exceeded one standard deviation of the background amount. This can be refined to improve the results beyond those given in the paper, by calculating thresholds that optimize the performance during a training period corresponding to a subset of the data, before then calculating the performance out-of-sample. While a brief report of preliminary results was presented at an earlier conference [33], the specific outcomes obtained are presented in the first part of the next section of this paper. The second approach that we took to choose subsets of words and suitable volume measures, employed an entirely automated scheme in which a Support Vector Machine (SVM) was used to select the features. Further details are given in the Appendix.

Fifth, in order to deduce the best keywords and the best search quantities (e.g. Google Trends volume and rate-of-change) to detect on-street protests, we needed to have a measure of success when comparing these quantities with the actual GSR record of protests within each country. The measure S0 that we used to define ‘best’ was given by the combination of three complementary quantities: accuracy B1 which is given by the sum of the number of true positives (TP) and true negatives (TN) divided by the sum of true positives (TP), true negatives (TN), false positives (FP) and false negatives (FN); the sensitivity P1 which is given by the number of true positives (TP) divided by the sum of true positives (TP) and false negatives (FN); the specificity P0, given by the number of true negatives (TN) divided by the sum of true negatives (TN) and false positives (FP). Hence

$$\begin{aligned}& \mathrm{B}1= \frac{\mathrm{TP}+\mathrm{TN}}{ \mathrm{TP}+\mathrm{TN}+\mathrm{FP}+\mathrm{FN}}:\quad \text{Accuracy}, \\& \mathrm{P}1= \frac{\mathrm{TP}}{ \mathrm{TP}+\mathrm{FN}}:\quad \text{Sensitivity}, \\& \mathrm{P}0= \frac{\mathrm{TN}}{ \mathrm{TN}+\mathrm{FP}}:\quad \text{Specificity}, \end{aligned}$$

which gives the overall performance measure

$$\begin{aligned}& \mathrm{S}0=\mathrm{B}1+\mathrm{P}1+\mathrm{P}0:\quad \text{Overall performance}. \end{aligned}$$

We then ran automatically all possible combinations of the pre-selected keywords and in each case we calculated values for the accuracy, sensitivity and specificity - and hence the overall measure S0. The maximum possible value of S0 can be seen to be 3 and the minimum is 0.

**Figure 1:**
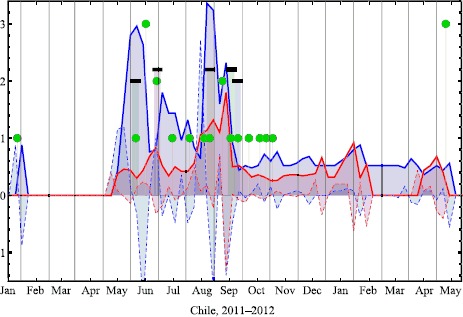
**Online Google Trend search volume and momentum vs. on-street protests for Chile.** Example shown is typical of results emerging from our analysis for countries across Latin America. Vertical axis shows normalized Google Trend search volume relative to average background values, for the words *protesta* (protest) in blue and *huelga* (strike) in red. Also shown as dashed lines is the momentum for each of these keywords, i.e. rate-of-change of these keyword search volumes. Green solid circles show the actual number of on-street protests in Chile. These take the values 1, 2, etc. but for clarity, no green solid circle is shown on days for which there are zero protests. Black dots, which are offset vertically for clarity, show days on which Google Trends volume and momentum for these keywords all rise above the background steady-state value (i.e. signal exceeds noise) and hence our analysis predicts an on-street protest in Chile. There appear to be rises in the volumes and momenta around the same time as bursts of actual on-street protests.

**Figure 2:**
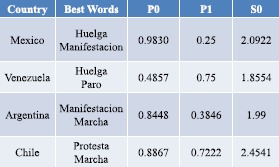
**Example of results across countries.** Chile happens to show the best performance of the semi-automated prediction scheme discussed in the text, i.e. highest S0. The value of B1 is not shown since $\mathrm{S}0=\mathrm{B}1+\mathrm{P}1+\mathrm{P}0$, hence B1 can be deduced using $\mathrm{B}1=\mathrm{S}0-\mathrm{P}1-\mathrm{P}0$.

**Figure 3:**
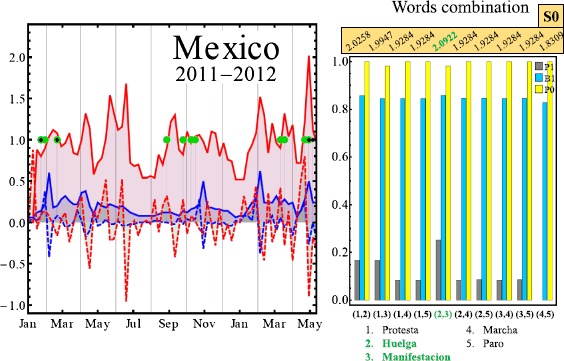
**Google Trends volume and momentum vs. on-street protests for Mexico.** Left panel: Green dots represent actual on-street civil unrest events. The black dots show predictions using our semi-automated procedure as discussed in the text. Solid red line shows volume for keyword *manifestacion* (i.e. manifestation) while dashed red line shows its momentum (i.e. rate of change of volume). Solid blue line shows volume for keyword *huelga* (i.e. strike) while dashed blue line shows its momentum (i.e. rate of change of volume). An accurate prediction corresponds to a coincident black and green dot, and corresponds to a true positive (TP). See text. Solid circles take the value of 1 or 0, but for clarity, no solid circle appears on days for which there are zero events. Right panel: Results for different pairs of keywords: (1) *protesta*, (2) *huelga*, (3) *manifestacion*, (4) *marcha*, (5) *paro*. Corresponding P1 (grey), B1 (cyan) and P0 (yellow) are shown for each combination. Adding these gives the S0 value given in the text. The maximum value is shown in green and corresponds to the optimal value of the combination S0, rather than the optimal value of B1 or P1 or P0 individually.

**Figure 4:**
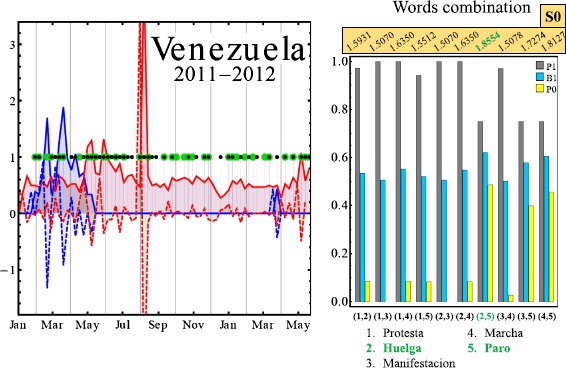
**Google Trends volume and momentum vs. on-street protests for Venezuela.** Left panel: Green dots represent actual on-street civil unrest events. The black dots show predictions using our semi-automated procedure as discussed in the text. Solid red line shows volume for keyword *paro* (i.e. stoppage) while dashed red line shows its momentum (i.e. rate of change of volume). Solid blue line shows volume for keyword *huelga* (i.e. strike) while dashed blue line shows its momentum (i.e. rate of change of volume). An accurate prediction corresponds to a coincident black and green dot, and corresponds to a true positive (TP). See text. Solid circles take the value of 1 or 0, but for clarity, no solid circle appears on days for which there are zero events. Right panel: Results for different pairs of keywords: (1) *protesta*, (2) *huelga*, (3) *manifestacion*, (4) *marcha*, (5) *paro*. Corresponding P1 (grey), B1 (cyan) and P0 (yellow) are shown for each combination. Adding these gives the S0 value given in the text. The maximum value is shown in green and corresponds to the optimal value of the combination S0, rather than the optimal value of B1 or P1 or P0 individually.

To obtain the predictions and hence black dots in Figures 1, 3 and 4, and hence the S0 values shown in Figures 2-4, we proceeded as follows within a given country. Suppose we are at given timestep *t*. Using the pair of words identified as having the highest S0 during the training period, we calculated the Google Trends volume at that timestep for this pair of keywords, and the momentum of this pair of keywords given by the change in search volume between timestep $(t-1)$ and *t*. We look to see if these two measures rise above the threshold values corresponding to background noise. If they do, we interpret this as a prediction of a likely on-street protest event in the next timestep $(t+1)$. Then we use the GSR database for actual protests to determine whether an on-street protest event actually took place (green dots). If it did, this is then counted as a true positive prediction (TP). If it did not, it is counted as a false positive (FP). At a timestep t where the volume and momentum did not both rise above the background noise value, this is a prediction of no protest event at timestep $(t+1)$. If according to the GSR an on-street protest actually did take place, this represents a false negative (FN). If it did not, it is a true negative (TN). In other words, an accurate prediction is shown by a coincident black and green dot, representing a true positive (TP). A green dot with no coincident black dot represents a false negative (FN) since no event was predicted but one did occur. A black dot with no coincident green dot represents a false positive (FP) since an event was predicted but did not occur. No black or green dot at a given timestep represents a true negative (TN) since no event was predicted and none occurred. When a black dot appears at the same position where a green dot is located, the TP score is increased by one, and similarly for FN, FP and TN. This procedure was then continued throughout the period of study, moving forward one timestep at a time. The same approach was implemented for real-time predictions during the IARPA project [31] using one timestep as one week. While it is not our purpose here to compare this present method to all other methods being presented in recent years for extracting predictions of future unrest from online chatter and content, we regard it as complementary to other such techniques. It remains an interesting future project to compare all such prediction schemes to see which works best under what conditions, and with what specific data.

Although the above methodology will be shown in the next section to produce some encouraging results, it has of course various limitations. First, in order to identify a signal and hence predict a protest, our method requires the search volume and rate-of-change (i.e. momentum) to be larger than the background noise level as described above. Hence we expect that our method will predict larger protests more reliably than smaller ones. Most of the events in the GSR are indeed larger protests, by reason of the fact that in order to be reported in the media and hence end up in the GSR, they must be noticeable in some way (e.g. >0.05% of the population). However we also note that the media do occasionally report an event that involves relatively few people but in which one member did something extreme such as inflicting visible damage on a particular landmark building, or throwing objects at a well-known politician. Since inclusion in the GSR involves no particular constraint about the size of protests, such smaller events will get included if they appeared in the media. The appearance of such additional events makes the prediction challenge facing our method even harder, and mean that the prediction results reported in this paper may underestimate the actual number of GSR events. It will be interesting to revisit the performance ratings shown in the next section when data becomes available concerning more precise numbers of protestors, e.g. if protestors start uploading reliable images of the crowd to a freely available online source. Second, our method is actually attempting to detect an expression of intent to protest - however many things can then prevent that protest from actually happening, e.g. strong police presence or threats of retaliation, or simply a resolution of the underlying issue. This would make our method likely overestimate the actual number of GSR events. Third, in regions where accessibility to the Internet is limited such as very poor and/or remote areas of a country, there may not be enough users to generate a measurable signal, which means we would not predict a GSR event and hence would miss any potential protest and underestimate the actual number of GSR events. Fourth, a positive signal predicting a protest may originate from users in neighboring areas - hence although the detection of the intent is real, they are not all located in the same place and hence do not generate a GSR event. Hence our method would overestimate the actual number of GSR events. Fifth, the keywords can change with time, region and space. Future work will try to unravel these limitations, though we note that several of them are expected to work in opposite directions in terms of the number of GSR events predicted, and so there may be some degree of cancellation.

## Results and discussion

Even though the GSR database of protests is as close to ground truth as could be obtained, a lack of specificity in the information available often made it hard for the IARPA team experts to tell whether events were actually spread over continuous days including weekends, or whether they were only on workdays. It was also sometimes hard to determine the exact location, since protests may only be reported as being outside the capital. Furthermore, it was often hard to tell the precise motive for the protest. For these reasons, we limit ourselves here to the following proof-of-concept: We show results of the application of our method in which we aggregate events to the level of the country, we aggregate over motives for the protest, and we aggregate events to the weekly scale, i.e. the timestep in our method is one week. We note that this means that our predictions are also at the weekly scale, i.e. our method is predicting events in the upcoming week as opposed to the next day.

Irrespective of whether we chose the initial pairings using the semi-automated or Support Vector Machine (SVM) approaches, it became clear that the most prominent words contributing to high Google Trends volume count and also high performance measure S0, were the five words *protesta* (i.e. protest), *huelga* (i.e. strike), *manifestacion* (i.e. demonstration), *marcha* (i.e. march) and *paro* (i.e. stoppage). It also became clear that pairs of these words could, if chosen correctly, yield high S0 values that could only marginally be improved upon using the SVM. We explain this by the fact that while one of these words alone in a story might correspond to a protest event, there may be many cases of false alarms due to another meaning of that word that is unrelated to on-street protests - but having two of these words from the list drastically reduces such false alarms. At the same time, having three keywords does not reduce this false alarm rate significantly more, but instead generates a far more complex space in which the search for maximum S0 has to occur. In short, we found that pairs of keywords from this subspace of five words provided a sufficiently rich space in which S0 could achieve a respectfully high value. At the same time, the richness of cultural nuances and motives for protest was captured by different pairs of words optimizing S0 for each country in turn. For example, searches related to protests in Venezuela were full of associations with mobilization related to electoral periods, while in Chile the most salient issue resulted from education reform.

Figure 1 illustrates the variation in Google Trends search volume and momentum for an example pair of keywords, in the representative case of Chile. As can be seen, there is the visual suggestion that a large change in the search volume and momentum are associated with the appearance of an on-street protest. This was our motivation for developing predictions of when on-street protests will occur. Figure 2 illustrates the performance of our semi-automated prediction method discussed above, for different pairs of keyword search volumes and momentum, as compared to real on-street protests for four major countries, together with the highest S0 obtained. The optimal pair of keywords in each case may not be the one that generates the highest possible values for B1, P1 and P0 individually, but it is the one that gives the highest performance in terms of S0 score. Since $\mathrm{S}0=\mathrm{B}1+\mathrm{P}1+\mathrm{P0}$, it follows that $\mathrm{B}1=\mathrm{S}0-\mathrm{P}1-\mathrm{P}0$ and hence B1 is not shown. Figures 3 and 4 show explicitly the examples for Mexico and Venezuela which are actually among our worst performing cases. The predictions for our semi-automated prediction method are shown as black dots in the figure, while the green dots in the figure stand for actual on-street protests from the GSR database, with the TP, TN, FP and FN values being counted as described in the previous section.

The cases of Mexico and Venezuela, which are shown in Figures 3 and 4, have the following measures of success. For Mexico: the number of false negatives $\mathrm{FN} = 9$; number of true positives $\mathrm{TP} = 3$; number of false positives $\mathrm{FP}=1$; number of true negatives $\mathrm{TN}=58$. Hence $\mathrm{B}1=0.859$, $\mathrm{P}0=0.983$, $\mathrm{P}1=0.25$ and so $\mathrm{S}0=\mathrm{B}1+\mathrm{P}1+\mathrm{P}0=2.09$. The right panel of Figure 3 shows the success measure values for different keyword pairs chosen from (1) *protesta*, (2) *huelga*, (3) *manifestacion*, (4) *marcha*, (5) *paro*. We find significant differences in performance as we change the pair of key words, as shown in Figures 3 and 4. To highlight this keyword dependence, we assign a color to the bar chart associated with the score contributions as follows: P1 (gray), B1 (cyan) and P0 (yellow). Combining these gives the S0 value shown. The pair yielding the highest S0, i.e. overall success, is $(2,3)$ corresponding to the pair of words *huelga* and *manifestacion* which are used to obtain the results in the left panel and hence 2.09, other combination yield to smaller score values. For Venezuela in Figure 4, the on-street events have a very different pattern from the case of Mexico, which suggests that a prediction model developed for one country based on transferring directly the pattern of activity in another, is likely to be highly inaccurate. The measures of success in Figure 4 for Venezuela, which is our worst performing case, are: number of false negatives $\mathrm{FN}=9$; number of true positives $\mathrm{TP}=27$; number of false positives $\mathrm{FP}=18$; number of true negatives $\mathrm{TN}=17$. Hence $\mathrm{B}1=0.620$, $\mathrm{P}0=0.486$, $\mathrm{P}1=0.75$ and so $\mathrm{S}0=\mathrm{B}1+\mathrm{P}1+\mathrm{P}0=1.86$. We believe that Venezuela is our poorest performer because protests tended to occur on a regular basis every Sunday due to upcoming elections - however we stress that its S0 value is still closer to the maximum of 3.0 than the minimum of 0. The right panel of Figure 4 shows the success measure values for different keyword pairs chosen from (1) *protesta*, (2) *huelga*, (3) *manifestacion*, (4) *marcha*, (5) *paro*. The pair of keywords yielding the highest S0, i.e. overall success, is $(2,5)$ corresponding to the pair of words *huelga* and *paro* which are used to obtain the results in the left panel and hence $\mathrm{S}0=1.86$.

We performed a comparison between our method and a null model in which the GSR events are shuffled over the same time period (17 months). This maintains the number of GSR events but randomizes their date of occurrence. Our method outperforms the null model result by more than fifteen standard deviations in countries like Chile where the main topics of protests are related to education, and hence social media is a trustworthy indicator of activities within the student population. For the case of Chile, a z-score of 15.64 results from a comparison with the null model. Results for other countries can vary, but our model is consistently better than the null model result.

In these figures, there are some peaks that occur in the search volume behavior for which no real event subsequently occurred, and likewise there are occasions where no peak occurs just before a real event. There are several potential reasons for this as we discussed earlier concerning the limitations, e.g. searches originating elsewhere in the same country for the first case and a lack of Internet access in the second case. Likewise there are small peaks that do not produce a prediction of a protest because the signal was below the threshold, and yet a real event occurred. Performing forensics on the case in Chile in January 2011, for example, we see that there was insufficient search activity for one of the key words and that new key words had come into play. Performing this type of forensic analysis at the individual event level over extended periods of many years, should allow an improvement of the methodology. For example, it may turn out that different words are needed at different times of the year or at different points in a business cycle or election cycle. Our method’s performance decreases as we increase the number of timesteps over which the future prediction is made. For example, we find a drop in the overall score of nearly 35% when predictions are generated two timesteps (i.e. two weeks) in advance when compared to one. Overall, the difference in the final score for the different countries that we have presented here, suggests that the implementation of our methodology in this paper needs to be carefully tailored to each specific country even when accurate data is available and it is geographically accurate.

Interestingly, the results from the Support Vector Machine approach were quite similar to the semi-automated approach discussed above. The Support Vector Machine approach also showed a similar preference for picking pairs of keywords, as opposed to much larger subsets for which the search process became more complex and hence involved significant amounts of computational effort, with the corresponding insight gained being far less. We therefore find the semi-automated method to be more desirable in practical terms.

## Conclusions

On-street protests result from complex, time-varying interactions between individuals, groups or populations within a given cultural and socioeconomic setting - and are therefore likely affected by feedback between online and on-street activity. This is reflected in our findings. In particular, we show that examination of online Google Trends search volumes and momenta around pairs of certain keywords can give signals that may pre-empt the appearance of on-street protests. Although it is known that Google Trends has changed its volume search quantifications, our methodology may be modified to meet new forms of volume measure so that our proof-of-principle remains valid. We also found that a more complex and less transparent approach using a Support Vector Machine to discover suitable combinations of keywords etc. did not significantly out-perform the simple, semi-automated approach.

Our work opens up the possibility of such predictions down to the urban level, using newer technology that connects online searches to local IP addresses and hence the likely location of the highest searches. A further feature of our approach is that it does not require answering the thorny social science question of why on-street protests occur, or why individuals are motivated to participate in particular protests.

## Figures and Tables

**Figure 5:**
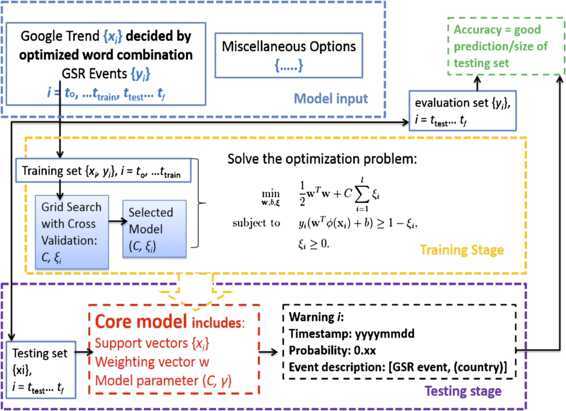
**Support Vector Machine flow diagram.** The input is the time-series of Google Trend search volumes $\{x_{i}\}$. There are multiple options which increase the complexity of the Support Vector Machine and hence decrease its transparency. However, its results and prediction performance are not significantly better than the semi-automated scheme that forms the main portion of this paper and whose results are presented in Figures 1-4.

## Appendix

Support Vector Machine (SVM): An SVM is a machine-learning mechanism for solving pattern recognition problems. In the method, the machine maps the features of data into a high dimensional input space where the number of dimensions equal the number of features, and constructs an optimal hyperplane separating those input points in this space. Different optimizing parameters generate various types of core function of the SVM. Figure 5 shows a flow diagram of the SVM used in the present paper. There are multiple options which increase the complexity of the Support Vector Machine and hence decrease its transparency. However, its results and prediction performance are not significantly better than the semi-automated scheme that forms the main body of results in this paper, as presented in Figures 1-4.

## References

[1] Mungiu-Pippidi A, Munteanu I (2009). Moldova’s “Twitter”. Revolut J Democr.

[2] Morozov E (2009). Iran: downside to the “Twitter Revolution”. Dissent.

[3] Barrat A, Barthelemy M, Vespignani A (2008). Dynamical processes on complex networks.

[4] Alvarez R, Garcia D, Moreno Y, Schweitzer F (2015). Sentiment cascades in the 15M movement. EPJ Data Sci.

[5] Garcia D, Garas A, Schweitzer F (2015). The language-dependent relationship between word happiness and frequency. Proc Natl Acad Sci.

[6] Dodds PS, Danforth CM (2009). Measuring the happiness of large-scale written expression: songs, blogs, and presidents. J Happiness Stud.

[7] Preis T, Reith D, Stanley E (2010). Complex dynamics of our economic life on different scales: insights from search engine query data. Philos Trans R Soc Lond Ser A.

[8] Preis T, Moat HM, Stanley E (2013). Quantifying trading behavior in financial markets using Google Trends. Sci Rep.

[9] Valenzuela S, Arriagada A, Scherman A (2012). The social media basis of youth protest behavior: the case of Chile. J Commun.

[10] Bennett WL, Segerberg A (2012). The logic of connective action: digital media and the personalization of contentious politics. Inf Commun Soc.

[11] Rainie H, Rainie L, Wellman B (2012). Networked: the new social operating system.

[12] González-Bailón S, Borge-Holthoefer J, Moreno Y (2012). Broadcasters and hidden influentials in online protest diffusion. Am Behav Sci.

[13] Onnela JP, Reed-Tsochas F (2010). Spontaneous emergence of social influence in online systems. Proc Natl Acad Sci.

[14] Morales AJ, Losada JC, Benito RM (2012). Users structure and behavior in an online social network during a political protest. Physica A.

[15] Ginsberg J, Mohebbi MH, Patel SR, Brammer L, Smolinski MS, Brilliant L (2009). Detecting influenza epidemics using search engine query data. Nature.

[16] Preis T, Moat HM (2014). Adaptive nowcasting of influenza outbreaks using Google searches. R Soc Open Sci.

[17] Guzik K (2009). Discrimination by design: data mining in the United States’ ‘War on Terrorism’. Surveill Soc.

[18] Liu S, Perra N, Karsai M, Vespignani A (2014). Controlling contagion processes in activity driven networks. Phys Rev Lett.

[19] Rahmandad H, Sterman J (2008). Heterogeneity and network structure in the dynamics of diffusion: comparing agent-based and differential equation models. Manag Sci.

[20] Centola D, Macy M (2007). Complex contagions and the weakness of long ties. Am J Sociol.

[21] Dodds PS, Muhamad R, Watts DJ (2003). An experimental study of search in global social networks. Science.

[22] Braha D (2012). Global civil unrest: contagion, self-organization, and prediction. PLoS ONE.

[23] Macy M (1991). Chains of cooperation: threshold effects in collective action. Am Sociol Rev.

[24] Siegel DA (2009). Social networks and collective action. Am J Polit Sci.

[25] Singer BD (1992). Mass media and communication processes in the Detroit riot of 1967. Public Opin Q.

[26] Biggs M (2005). Strikes and forest fires: Chicago and Paris in the late nineteenth century. Am J Sociol.

[27] Biggs M (2003). Positive feedback in collective mobilization: the American strike wave of 1886. Theory Soc.

[28] Berman E, Laitin D (2008). Religion, terrorism and public goods: testing the club model. J Public Econ.

[29] Lichbach MI (1992). Nobody cites nobody else: mathematical models of domestic political conflict. Def Peace Econ.

[30] Davies J (2002). Narratives and social movements: the power of stories. Stories of change: narrative and social movements.

[31] Matheny J (2013) Test and evaluation in ACE and OSI IARPA. http://semanticommunity.info/@api/deki/files/21696/3-ACE_and_OSI_NIST_Brief.pdf. See also: http://www.iarpa.gov/index.php/research-programs/osi

[32] Cadena J, Korkmaz G, Kuhlman CJ, Marathe A, Ramakrishnan N, Vullikanti A (2015). Forecasting social unrest using activity cascades. PLoS ONE.

[33] Manrique P, Qi H, Morgenstern A, Velasquez N, Lu TC, Johnson NF (2013). Context matters: improving the uses of big data for forecasting. IEEE intelligence and security informatics.

